# Effectiveness of early discharge planning in acutely ill or injured hospitalized older adults: a systematic review and meta-analysis

**DOI:** 10.1186/1471-2318-13-70

**Published:** 2013-07-06

**Authors:** Mary T Fox, Malini Persaud, Ilo Maimets, Dina Brooks, Kelly O’Brien, Deborah Tregunno

**Affiliations:** 1 School of Nursing, York University, 4700 Keele Street, Toronto, Ontario M3J 1P3, Canada; 2 Steacie Science and Engineering Library, York University, 4700 Keele Street, Toronto, Ontario M3J 1P3, Canada; 3 Department of Physical Therapy and Graduate Department of Rehabilitation Sciences, University of Toronto, 27 Kings College Circle, Toronto, Ontario M5S 1A1, Canada

**Keywords:** Discharge planning, Aged, Length of stay, Hospital readmission, Patient discharge, Systematic review, Meta-analysis

## Abstract

**Background:**

Older age and higher acuity are associated with prolonged hospital stays and hospital readmissions. Early discharge planning may reduce lengths of hospital stay and hospital readmissions; however, its effectiveness with acutely admitted older adults is unclear.

**Methods:**

In this systematic review, we compared the effectiveness of early discharge planning to usual care in reducing index length of hospital stay, hospital readmissions, readmission length of hospital stay, and mortality; and increasing satisfaction with discharge planning and quality of life for older adults admitted to hospital with an acute illness or injury.

We searched the Cochrane Library, DARE, HTA, NHSEED, ACP, MEDLINE, EMBASE, CINAHL, Proquest Dissertations and Theses, PubMed, Web of Science, SciSearch, PEDro, Sigma Theta Tau International’s registry of nursing research, Joanna Briggs Institute, CRISP, OT Seeker, and several internet search engines. Hand-searching was conducted in four gerontological journals and references of all included studies and previous systematic reviews. Two reviewers independently extracted data and assessed risk of bias. Data were pooled using a random-effects meta-analysis. Where meta-analysis was not possible, narrative analysis was performed.

**Results:**

Nine trials with a total of 1736 participants were included. Compared to usual care, early discharge planning was associated with fewer hospital readmissions within one to twelve months of index hospital discharge [risk ratio (RR) = 0.78, 95% CI = 0.69 − 0.90]; and lower readmission lengths of hospital stay within three to twelve months of index hospital discharge [weighted mean difference (WMD) = −2.47, 95% confidence intervals (CI) = −4.13 − −0.81)]. No differences were found in index length of hospital stay, mortality or satisfaction with discharge planning. Narrative analysis of four studies indicated that early discharge planning was associated with greater overall quality of life and the general health domain of quality of life two weeks after index hospital discharge.

**Conclusions:**

Early discharge planning with acutely admitted older adults improves system level outcomes after index hospital discharge. Service providers can use these findings to design and implement early discharge planning for older adults admitted to hospital with an acute illness or injury.

## Background

Individuals who present with older age and higher levels of acuity are at risk for longer hospital stays and hospital readmission [1]. Prolonged hospital stays and hospital readmissions are costly to both older adults and the healthcare system. These events are associated with increased risk of iatrogenic complications, functional decline, and mortality in older adults [2] as well as increased hospital expenditures. Older adults account for 50% of hospital expenditures in Canada [3] and 45% of hospital expenditures in the United States (US) [4] despite representing only 14% of the Canadian population [5] and 13% of the US population [6]. With fiscal restraints and projected increases in age demographics in several countries, reducing lengths of hospital stay and hospital readmissions for older adults is a priority to healthcare service-providers and policy-makers [7-9]. While Canadian and US healthcare providers have either adopted or are considering various discharge planning programs [10-13], the overall effect of discharge planning introduced during the acute phase of an older person’s illness or injury, is unclear and unquantified.

Early discharge planning is defined by interventions initiated during the acute phase of an illness or injury to facilitate transition of care back to the community as soon as the acute event is stabilized [14]. Prior reviews examining the effectiveness of discharge planning in reducing lengths of hospital stay [15-20] and hospital readmissions [15-20] have several limitations, supporting the need for this current review. All prior reviews combined data from studies that included younger and older adults [15-20]. Three of these reviews also combined data from studies that initiated discharge planning during the acute and post-acute phases of illness or injury [16,19,20], including at the time of hospital discharge or later [15,16,20]. The results may not be generalizable to hospitalized older adults in the acute phase of an illness or injury.

The objective of this study was to compare the effectiveness of early discharge planning to usual care primarily in reducing index length of hospital stay, hospital readmissions, and readmission length of hospital stay and secondarily in reducing mortality and increasing satisfaction with discharge planning and quality of life for older adults admitted to hospital with an acute illness or injury.

## Methods

We conducted a systematic review that compared early discharge planning, initiated during the acute illness or injury phase, to usual care using the Cochrane Collaboration Protocol [21].

### Eligibility criteria

Eligible studies included published and unpublished randomized control and quasi-experimental trials with parallel controls that compared early discharge planning to usual care for adults aged 65 years and older in the acute illness or injury phase, defined as “the period during which an illness or injury is being intensively treated and stabilized” (p. xii) [22]. Early discharge planning was defined by interventions during the acute phase of illness or injury to facilitate transition of care back to the community [14]. Usual care was defined as any care in which discharge planning, if provided, was not identified as having been initiated early, during the acute phase of illness or injury.

Eligible studies included at least one primary outcome (index length of hospital stay, hospital readmissions, or readmission length of hospital stay) or at least one secondary outcome (mortality, satisfaction with discharge planning, or quality of life). Index length of hospital stay was defined as the total number of consecutive days in the study hospital where early discharge planning or usual care was initiated. Hospital readmissions refer to the number of patients readmitted one or more times to an acute care hospital between index hospital discharge (regardless of discharge destination) and the end of study follow-up. When study authors defined hospital readmissions by rehospitalization or death after index hospital discharge [23] we presumed that patients who died after index hospital discharge also experienced hospital readmission. Readmission length of hospital stay refers to the mean number of hospital days per patient from the time of index hospital discharge to the end of study follow-up. Mortality was defined as the cumulative number of deaths from index hospital admission to the end of study follow-up. Satisfaction with discharge planning was defined by the level of satisfaction with discharge planning that included satisfaction with hospital communication and/or co-ordination and continuity of care across settings as reported by each of three groups: older adults, caregivers, and community healthcare providers. Quality of life refers to level of well-being as reported by each of two groups: older adults and their caregivers.

Ineligible studies were those that were unavailable in English or French; compared usual care units to acute care for elders units (ACE) or geriatric units which provided early discharge planning as one of two or more ACE intervention components; compared usual care to exercise programs in which early discharge planning was provided; included historical control groups; included patients in the sub-acute or post-acute phase, which refer to the period “following stabilization of a disease or injury” (p. xii) [22]; included social admissions; or included patients receiving palliative care or admitted for elective surgical procedures such as arthroplasty. Studies that initiated the intervention upon or after index hospital discharge, or studies that focused on the provision of care after index hospital discharge were also excluded.

### Search strategy and study selection

The literature search was conducted by an information specialist with input from team members with expertise in the clinical area to identify keywords. Keywords included, but were not limited to: discharge planning, comprehensive discharge planning, early discharge planning, early supported discharge, transition, aftercare, patient care planning, advance care planning, length of stay, patient readmission, patient transfer, and patient care management (Additional file 1). Electronic databases searched included: EBM Reviews consisting of the Cochrane Library, DARE, HTA, NHSEED and ACP; MEDLINE; EMBASE; CINAHL; Proquest Dissertations and Theses; PubMed; Web of Science; SciSearch; PEDro; Sigma Theta Tau International’s registry of nursing research; Joanna Briggs Institute; CRISP; and OT Seeker. Internet search engines included: Google, Yahoo, Scirus, Healia, and HON. Hand-searching was conducted in The Gerontologist, Age and Ageing, Journal of the American Medical Association, Journal of the American Geriatrics Society, and bibliographies of all included studies and previous systematic reviews. We also searched for specific programs including Care Transitions, Transitional Care, Project BOOST Society of Hospital Medicine, Re-engineered Discharge and Transforming Care at the Bedside in EBM Reviews; MEDLINE; EMBASE; CINAHL; PubMed; and Web of Science (Additional file 2).

Teams of two reviewers from the group of investigators independently screened abstracts of the retrieved citations for potential inclusion. For the search on specific programs, one reviewer screened the abstracts and a second reviewer screened the abstracts that contained the first reviewer’s notes concerning the abstracts’ eligibility. Disagreements about the eligibility of articles were resolved by discussion and consensus between two reviewers. When necessary, the complete article was reviewed to determine eligibility. Where consensus could not be reached, a third team member independently reviewed the abstract or complete article and determined final inclusion.

### Data extraction & risk of bias assessment

Two reviewers independently extracted relevant data from each included article and entered the data into a standardized pilot tested data extraction form. Information categories included: study design, participant characteristics, setting, health care providers, early discharge planning or usual care intervention elements, occasions of measurement, and outcomes. Two reviewers independently assessed the risk of bias of each study using six defined domains: (1) sequence generation, (2) allocation concealment, (3) blinding of participants, personnel, and outcome assessors, (4) completeness of outcome data, (5) selective reporting, and (6) other sources of bias [21].

Study authors were contacted if additional data were required. Disagreements on data extraction and risk of bias assessments were resolved by consensus with the assistance of a third team member when necessary. Consensus data from included studies were entered into Review Manager (RevMan, version 5.1) computer software, using the double-entry option [24].

### Data analysis

Where we had sufficient data and where studies were comparable in terms of outcomes, we performed meta-analyses using RevMan [24]. Continuous and dichotomous outcomes were analyzed using a random effects model to calculate a weighted mean difference (WMD) and risk ratio (RR) respectively, with 95% confidence intervals (CI). A *P*-value < 0.05 was considered statistically significant for an overall effect. A *P*-value < 0.10 was considered statistically significant for heterogeneity [25]. Degree of heterogeneity is reported by the I^2^ statistic which refers to the degree of variation across studies [21]. In situations where heterogeneity was statistically significant, sensitivity analyses were performed whereby studies were systematically removed from meta-analyses to determine robustness of findings [26]. Decisions for removing studies during sensitivity analyses were based on their potential source of variability - duration of outcome measurement. Studies that reported outcomes at one year were first to be removed, followed by studies that reported outcomes for successively shorter periods of time.

In situations where meta-analysis was not possible, narrative analyses were performed; and the proportion of studies which identified an overall effect for early discharge planning compared to usual care was reported. A *P*-value < 0.05 was considered statistically significant for an overall effect.

## Results

### Description of studies

**Figure 1:**
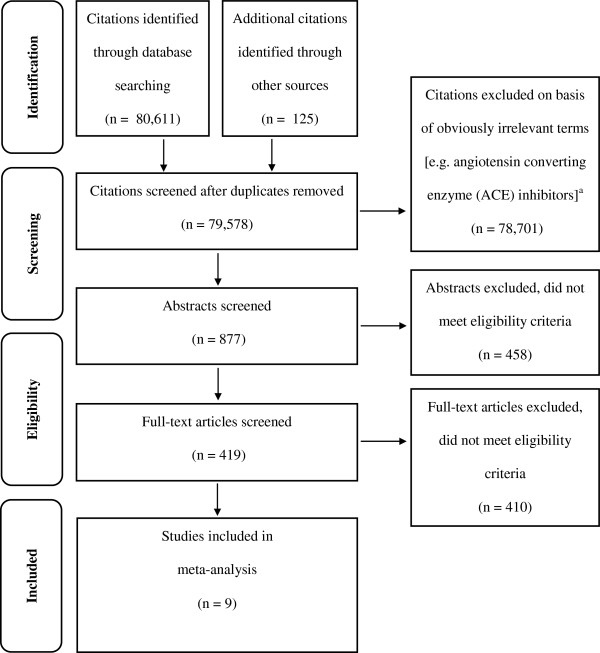
**PRISMA Flow Diagram **[35]**.**

Searches of all sources yielded 79,578 citations of which nine studies met the inclusion criteria (Figure 1) [23,27-34]. Characteristics of the nine studies are provided in Additional file 3: Table S1. A report of the review using the Preferred Reporting Items for Systematic Reviews and Meta-Analyses (PRISMA) statement guidelines [35] is provided in Additional file 4.

A total of 1736 participants were included in this review. The average study participant was 79 years of age and female (60%); was admitted to either a medical unit [23,31-34], an orthopedic unit [27,30], or an intensive care unit [29] for either the medical and/or surgical management of a cardiovascular illness (76%) or the surgical management of a hip fracture (18%); and presented with other co-morbidities including hypertension, diabetes mellitus, cancer and pulmonary diseases. The average participant was cognitively intact [23,28-32]. Most studies (67%) were conducted in the US [23,28,29,31-33]. Studies that provided information on living arrangements pre-index hospital admission reported most participants as living in the community [23,28,31-33] with family or significant others [27,28,31].

Early discharge planning was most often initiated by nurses [23,28-30] within 24 to 48 hours of index hospital admission (Additional file 5: Table S2) [23,27,29,30,33]. Early discharge planning involved: assessing the needs of older adults for discharge home with a focus on functional needs [23,28-32]; providing education to older adults and where available, to their families or caregivers [23,27,28,30-34]; reviewing and adjusting medications [23,31-34]; transferring information to successive in-hospital healthcare providers or coordinating care with community healthcare providers [23,28-34]; and following-up with one or more home visits and/or telephone calls after index hospital discharge [23,28,30-33].

Where described, usual care included unstructured routine or standard discharge planning provided by nurses or physicians [28,30-32] that was initiated post-operatively [27] or after transfer from intensive care units one to three days prior to index hospital discharge [29].

### Risk of bias

Risk of selection bias resulting from inadequate sequence generation was low in seven of the nine studies [23,28,29,31-34]. Two studies either provided insufficient information to draw conclusions in this domain [30] or were considered not to have been properly randomized [27].

Risk of selection bias resulting from inadequate allocation concealment was low in five of the nine studies [23,27,32-34]. In the other four studies, risk of bias was unclear because allocation information was not provided [28-31].

Risk of performance bias relating to double blinding was either unclear because seven studies did not provide sufficient information to draw conclusions in this domain [23,27,29-33] or high because of the absence of double blinding [34]. Only one study was double blinded [28].

Risk of detection bias relating to blinding of outcome assessors was unclear because five studies did not provide this information [27,29-32]. Only four studies were determined to have low risk of detection bias related to blinding of outcome assessors [23,28,33,34].

Risk of attrition bias related to completeness of outcome data was low in six studies [23,27,28,31-33] and high [29,34] or unclear [30] in three studies.

Risk of reporting bias due to selective reporting was low in almost all studies [23,27-30,32-34] except for one study which reported outcome data on quality of life for a subgroup of the study sample [31]. All nine studies did not appear to be at risk for other sources of bias that were not addressed in prior domains.

### Effectiveness of early discharge planning

**Figure 2:**
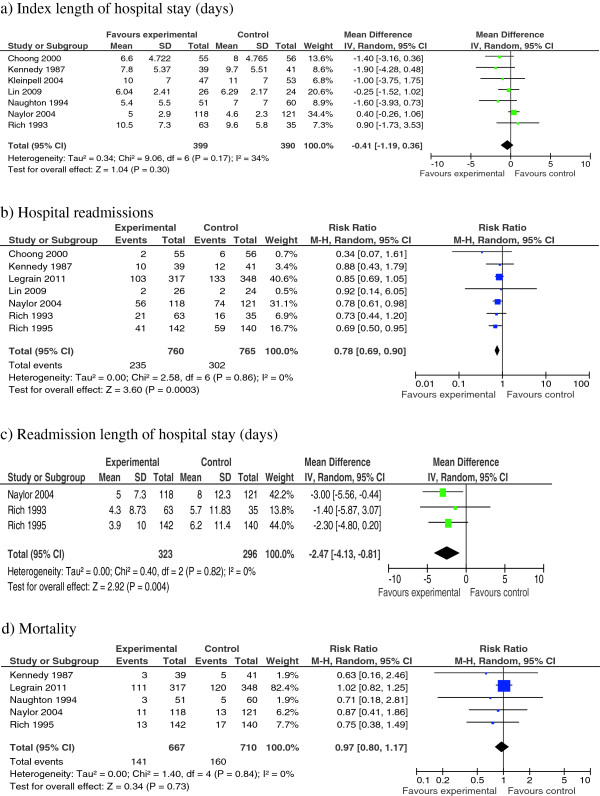
**Forest Plots. a)** Index length of hospital stay (days). **b)** Hospital readmissions. **c)** Readmission length of hospital stay (days). **d)** Mortality.

In total, four meta-analyses were performed for the following outcomes: index length of hospital stay, hospital readmissions, readmission length of hospital stay, and mortality (Figure 2). Sensitivity analyses were not performed because heterogeneity was not significant. Narrative analyses were performed for older adults’ satisfaction with discharge planning and quality of life because the two studies that reported satisfaction with discharge planning and the four studies that reported quality of life employed different outcome measurement scales or did not report baseline data. Neither meta-analyses nor narrative analyses could be performed for caregiver or community healthcare provider satisfaction with discharge planning or for caregiver quality of life because none of the studies reported on these outcomes.

#### Index length of hospital stay

Index length of hospital stay was reported in seven studies [23,27-30,32,33]. Meta-analysis of these seven studies identified no significant differences in older adults who received early discharge planning compared with those who received usual care (Figure 2a).

#### Hospital readmissions

Seven studies reported on hospital readmissions: within one month [27], two months [28], three [30-32], six [34], or twelve months of index hospital discharge [23]. Meta-analysis of these seven studies identified that older adults who received early discharge planning experienced significantly fewer hospital readmissions within one or twelve months of index hospital discharge (RR = 0.78, 95% CI = 0.69 − 0.90; *P* = 0.0003), when compared with those who received usual care (Figure 2b). This amounts to a reduction of 22% in hospital readmissions, favoring early discharge planning.

#### Readmission length of hospital stay

Three studies reported on readmission length of hospital stay within three months [31,32], or within twelve months [23] of index hospital discharge. Meta-analysis of these three studies identified that older adults who received early discharge planning experienced a lower readmission length of hospital stay of almost two and a half days (WMD = −2.47, 95% CI = −4.13 − −0.81; *P* = 0.004) when compared to usual care (Figure 2c).

#### Mortality

Five studies reported on mortality from index hospital admission to discharge [33] or within two months [28], three months [31], six months [34] or twelve months [23] of index hospital discharge. Meta-analysis of these five studies identified no significant difference in mortality from index hospital admission to within two to twelve months of index hospital discharge in older adults who received early discharge planning compared with those who received usual care (Figure 2d).

#### Satisfaction with discharge planning

Satisfaction with discharge planning was reported in two studies [23,30]. The two studies measured older adults’ satisfaction with discharge planning at two weeks after index hospital discharge using different Likert type scales developed by the study authors [23,30]. No differences were found in older adults’ satisfaction with discharge planning in either study.

#### Quality of life

Quality of life was reported in four studies [23,29-31]. Two studies measured specific domains of quality of life for older adults using the eight subscales of the SF-36 [29,30]; one study measured overall quality of life using the Minnesota Living with Heart Failure Questionnaire [23]; and one study measured overall quality of life using the Chronic Heart Failure Questionnaire [31]. Results from individual studies suggested that compared to usual care, older adults who received early discharge planning had significantly higher scores in overall quality of life at two weeks [23] and at three months [23,31] after index hospital discharge, as well as in the SF-36 domain of general health at two weeks after index hospital discharge [29,30]. In the two studies that used the SF-36, there were no consistent differences in the other seven domains of quality of life that included physical functioning, role limitations due to physical problems, bodily pain, mental health, role limitations due to emotional problems, social functioning, and vitality [23,31].

## Discussion

Results from meta-analyses demonstrate that, compared to usual care, early discharge planning initiated during the acute illness or injury phase has beneficial effects in reducing hospital readmissions and readmission length of hospital stay but no beneficial effects in reducing index length of hospital stay or mortality. Although further research is needed, results from narrative analyses suggest that early discharge planning may improve overall quality of life and the general health domain of quality of life in acutely ill or injured older adults. Given the demographic and health characteristics of the average study participant, findings are mainly applicable to cognitively intact older North Americans residing with their families or significant others in the community prior to index hospital admission for the management of cardiovascular illnesses and other co-morbidities.

### Implications for practice and policy

Our findings have relevance to clinicians, hospital administrators, and policy-makers. The findings suggest that by implementing early discharge planning focused on functional needs’ assessment for discharge home, patient and caregiver education and follow-up, medication review and information transmittal, clinicians may anticipate reductions in older adults’ hospital readmissions by 22% and readmission lengths of hospital stay by almost two and a half days, compared with usual care. These reductions may have significant resource implications to countries experiencing population aging, particularly Canada where the proportion of adults aged 65 and older is projected to increase to 25% in the next 23 years [7]. Hospital lengths of stay for older Canadians are one and a half times greater than those of younger adults [3]. With each 5-year increase in age after 65, per capita hospital costs rise from four to eleven times those of younger Canadians, and surpass 50% of all hospital expenditures [3]. These trends are expected to continue to the point where one out of three older Canadians will be at least 80 years old in 2036 [36]. Canadian hospitals and community health services, the two providers involved in an older person’s care transition, are funded independently of each other [37] and therefore may vary in their approach to discharge planning. In the US, discharge planning services are the responsibility of private sector purchasers and public payers of hospital and community health services, some of whom initiate discharge planning services for older adults only after their acute event has resolved, and they have returned home [38]. This review provides synthesized evidence to support the initiation of discharge planning early, while an older person’s acute illness or injury is being treated in hospital. Early discharge planning has the potential to significantly improve system efficiency by reducing hospital readmissions and readmission lengths of hospital stay in Canada and the US, as well as in other countries, such as China, where discharge planning services are reported to be limited [39].

### Comparison with previous research

The findings of our review are similar to the majority of prior reviews which evaluated discharge planning for younger and older adults using narrative analysis [18,19] or meta-analysis [17,20]. Prior reviews found discharge planning to be associated with fewer hospital readmissions [17,20], greater quality of life [17,19] but not reduced mortality [19,20]. However, unlike the majority of prior reviews which did not differentiate between index and readmission length of hospital stay and found inconclusive [19] or significant effects on overall length of hospital stay [20], our study identified significant reductions in readmission length of hospital stay but not in index length of hospital stay. The latter finding concurs with a recent descriptive component analysis which found early discharge planning to have a negligible effect size association with index length of hospital stay for acutely ill or injured older adults [40].

### Strengths and limitations of the review

Because this review included nine studies with limited information of study methods, we were limited in our ability to draw conclusions regarding level of bias in several domains. The number of studies included in meta-analyses ranged from three to eight and their combined samples sizes ranged from 619 to 1525 which may have influenced precision of the estimates. Heterogeneity was not significant in any of the meta-analyses, supporting validity of the results.

In four (44%) of the included studies, follow-up contact by way of in-home visits or telephone calls continued for three months after index hospital discharge. Consequently, the beneficial effects of early discharge planning on outcomes measured after index hospital discharge, including hospital readmissions, readmission lengths of hospital stay, and quality of life, may be related to the duration for which follow-up contact was continued into the community.

### Implications for future research

This review highlights the limited number of studies that examined the effectiveness of early discharge planning on outcomes important to older adults, their caregivers, and community healthcare providers; specifically satisfaction with early discharge planning and quality of life. The goal of early discharge planning is to facilitate transition of care back to the community; that is, back to the individuals responsible for resuming care after hospitalization. With increased focus on preventing hospital readmissions in older adults, future research should examine the effectiveness of early discharge planning on caregiver satisfaction and quality of life as well as community healthcare provider satisfaction.

This review also highlights the limited number of studies conducted with older adults with dementia. This subgroup of the older population has more than twice the rate of hospital admission [41,42] with lengths of hospital stays ranging from 1.4 [43] to 1.7 [42] times those of cognitively intact older adults. Older adults with dementia are also more vulnerable to experiencing functional decline and iatrogenic complications during hospitalization [44]. Moreover, dementia presents one of the most taxing illnesses for the health and well-being of caregivers, most of whom themselves are older [45]. Consequently, future research is needed to understand the effectiveness of early discharge planning on patient, caregiver and system level outcomes with this subgroup of the older adult population.

Lastly, the review indicates the limited number of studies conducted with older adults admitted with an acute injury or illness other than cardiovascular illnesses. In addition to cardiovascular illnesses, respiratory illnesses as well as acute injury such as a hip fracture are among the top reasons for an older person’s hospital admission [7,8,46]. Similarly, the review highlights the limited number of studies that compared the effectiveness of early discharge planning to usual care in countries other than the US. Future studies should examine the effect of early discharge planning with different subgroups of the older population, including those residing in countries where the contexts of care may be different than that in the US. Future updates of this review may enable us to incorporate new studies to determine the effectiveness of early discharge planning with different subgroups of the acutely admitted older adult population.

## Conclusion

Compared to usual care, early discharge planning, initiated during the acute phase of an illness or injury, reduces hospital readmissions and readmission lengths of hospital stay for older adults. Findings are predominantly applicable to older, cognitively intact North Americans living with their families or significant others in the community prior to index hospital admission for the management of cardiovascular illnesses. Early discharge planning does not appear to reduce index length of hospital stay or mortality, nor does it increase satisfaction with the discharge planning process itself in older adults when compared to usual care. Clinicians, administrators, and policy makers can use these findings to design and implement early discharge planning for older adults admitted to hospital with an acute illness or injury.

## Competing interests

The authors declare that they have no competing interest.

## Authors’ contributions

Study concept: MTF. Study design: MTF, IM, KO, DB, and DT. Literature searching and initial records screening: MTF, MP, and IM. Abstract and article screening for eligibility: MTF, MP, KO, DB, and DT. Data extraction and risk of bias assessments: MTF and MP. Data analysis: MTF, MP, and KO. Manuscript preparation: MF. Manuscript editing: IM, DB, KO, and DT. All authors read and approved the final manuscript.

## Pre-publication history

The pre-publication history for this paper can be accessed here:

http://www.biomedcentral.com/1471-2318/13/70/prepub

## Supplementary Material

Additional file 1 Search Strategy for MEDLINE(OVID). Click here for file

Additional file 2 Search Strategy on Specific Programs for MEDLINE(OVID). Click here for file

Additional file 3: Table S1 Descriptive characteristics of studies included in systematic review and meta-analysis. Notes: ^a^ Not all outcomes measured at all time points. Where outcomes were comparable, narrative analysis or meta-analysis performed; ^b^ Duration of follow-up post index hospital discharge. Abbreviations: CHF = congestive heart failure; HT = hypertension; MI = myocardial infarction; N = sample size; NR = not reported; RCT = randomized controlled trial; USA = United States of America. Click here for file

Additional file 4 PRISMA 2009 Checklist. Click here for file

Additional file 5: Table S2 Characteristics of early discharge planning intervention and usual care. Abbreviations: ADL = activities of daily living; APN = advanced practice nurse; CNS = clinical nurse specialist; GEM = geriatric evaluation and management; IADL = instrumental activities of daily living; ICU = intensive care unit; NR= not reported; PT = physical therapist; SW = social worker. Click here for file
