# Supplementary material for: Effectiveness of early discharge planning in acutely ill or injured hospitalized older adults: a systematic review and meta-analysis
Source: BMC Geriatr. 2013 Jul 6;13:70. doi: 10.1186/1471-2318-13-70 (PMC3707815; doi:10.1186/1471-2318-13-70)
Supplement: Additional file 1 — Search Strategy for MEDLINE(OVID). [file 1471-2318-13-70-S1.pdf]

Additional File 1. Search Strategy for MEDLINE(OVID) [a]

Limits applied:

Published Date: yr="1985 -Current" [b]

Age Groups: "all aged (65 and over)"

Languages: English or French

Organism: humans

Study Types: case reports or clinical trial, all or clinical trial or comparative study or controlled clinical trial or meta analysis or multicenter study or randomized controlled trial or "review"

A1 – Subject Heading Searches

Population:

aged/ or "aged, 80 and over"/ or frail elderly/ or Geriatrics/ or veterans/

Assessment and Aftercare:

Geriatric Assessment/ or Health Services for the Aged/ or aftercare/ or comprehensive health care/ or patient care planning/ or advance care planning/ or progressive patient care/ or patient care team/ or disability evaluation/ or patient education as Topic/ or Case Management/ or Critical Pathways/ or Program Evaluation/

OR Health Status/ or health/ [limit to yr="1985 - 1989"]

Hospital Units:

Hospital Units/ or Intensive Care Units/ or Emergency Medical Services/ or emergency service, hospital/ or trauma centers/ or hospitals/ or intensive care units/ or coronary care units/ or recovery room/ or respiratory care units/ or operating rooms/ or rehabilitation centers/ or General Surgery/ or medicine/ or emergency medicine/ or internal medicine/

or Hospitals, Veterans/ or Acute disease/ or Psychiatric Department, Hospital/ or Hospitals, Psychiatric/

Acute Conditions:

exp "bacterial infections and mycoses"/ or exp virus diseases/ or exp parasitic diseases/ or exp neoplasms/ or exp musculoskeletal diseases/ or exp digestive system diseases/ or exp stomatognathic diseases/ or exp respiratory tract diseases/ or exp otorhinolaryngologic diseases/ or exp nervous system diseases/ or exp eye diseases/ or exp male urogenital diseases/ or exp "female urogenital diseases and pregnancy complications"/ or exp cardiovascular diseases/ or exp "hemic and lymphatic diseases"/ or exp "congenital, hereditary, and neonatal diseases and abnormalities"/ or exp "skin and connective tissue diseases"/ or exp "nutritional and metabolic diseases"/ or exp endocrine system diseases/ or exp immune system diseases/ or exp "wounds and injuries"/ or exp "pathological conditions, signs and symptoms"/ or acute disease/ or exp "psychiatry and psychology (non mesh)"/ or exp Accidents/

Complications:

postoperative complications/ or pain/ or pain, intractable/ or pain, postoperative/ or shock, surgical/ or surgical wound infection/ or venous thrombosis/ or Accidental Falls/ or Immobilization/ or Restraint, Physical/ or hip fractures/ or femoral neck fractures/ or osteoporotic fractures/ or Cognition Disorders/ or Delirium/ or confusion/ or Cognition/ or Delirium, Dementia, Amnestic, Cognitive Disorders/ or Depression/ or Affect/ or polypharmacy/ or Drug Monitoring/ or mood disorders/ or skin ulcer/ or leg ulcer/ or pressure ulcer/ or Ulcer/ or malnutrition/ or protein deficiency/ or protein-energy malnutrition/ or Nutritional Status/ or Elder Nutritional Physiological Phenomena/ or

dehydration/ or starvation/ or wasting syndrome/ or Fecal Incontinence/ or Urinary Incontinence/ or Urinary Catheterization/ or Iatrogenic Disease/ or Cross Infection/ or catheter-related infections/ or pneumonia/ or soft tissue infections/ or wound infection/ or Urinary Tract Infections/ or Catheters, Indwelling/ or sleep disorders/ or sleep deprivation/ or sleep disorders, circadian rhythm/ or sleep disorders, intrinsic/ or "disorders of excessive somnolence"/ or "sleep initiation and maintenance disorders"/ or Dizziness/ or syncope/ or syncope, vasovagal/ or vertigo/ or Mobility Limitation/ or Hospital Mortality/ or Mortality/ or Vision, Low/ or Hearing Loss, Bilateral/ or Hearing Loss, Functional/ or Hearing Loss/

Cost and Cost Analysis:

"Costs and Cost Analysis"/ or Cost allocation/ or Cost-benefit analysis/ or Cost control/ or Cost savings/ or Health care costs/ or Hospital costs/

Rehabilitation:

Rehabilitation/ or "activities of daily living"/ or early ambulation/ or exp exercise therapy/ or occupational therapy/ or treatment outcome/ or physical therapy modalities/ or exercise movement techniques/ or breathing exercises/ or exercise therapy/ or motion therapy, continuous passive/ or muscle stretching exercises/ or resistance training/ or music therapy/ or recovery of function/ or exercise/ or Self care/ or medication therapy management/ or Nutrition therapy/ or diet therapy/ or nutritional support/ or enteral nutrition/ or Parenteral Nutrition/ or nutrition assessment/ or Nutritional Status/ or Fluid therapy/ or Accident prevention/ or safety/ or primary prevention/ or secondary prevention/ or tertiary prevention/ or Safety Management/ or Patient care/ or aftercare/ or critical care/ or intensive care/ or perioperative care/ or intraoperative care/ or

postoperative care/ or preoperative care/ or night care/ or patient positioning/ or perioperative nursing/ or emergency nursing/ or geriatric nursing/ or operating room nursing/ or postanesthesia nursing/ or rehabilitation nursing/ or "moving and lifting patients"/ or patient positioning/ or perioperative period/ or intraoperative period/ or postoperative period/ or preoperative period/ or Psychiatric Nursing/ or "Physical Therapy (Specialty)"/ or Physical Therapy Modalities/ or Occupational Therapy/ or Physical Medicine/ or Primary Nursing/ or Nursing Assessment/ or Nursing, Team/ or Nursing Process/ or Patient Care Planning/ or Social Work, Psychiatric/ or Social Work/ or Pharmacists/ or physicians/ or general practitioners/ or physicians, family/ or quality of health care/ or risk factors/ or Caregivers/ or Clinical Protocols/ or Therapeutics/ or Environment Design/ or "Interior Design and Furnishings"/ or Toilet Facilities/ or Self-Help Devices/ or Wheelchairs/ or Hearing Aids/ or Protective Devices/ or "Continuity of Patient Care"/ or exp Bandages/

OR Primary Health Care/ [limit to yr="1985 - 1990"]

Hospitalization and Discharge:

hospitalization/ or "length of stay"/ or patient admission/ or patient discharge/ or patient readmission/ or patient transfer/ or patient care management/ or patient-centered care/ or institutionalization/

Delivery of Healthcare:

"delivery of health care"/ or "delivery of health care, integrated"/ or "quality of health care"/ or "outcome and process assessment (health care)"/ or "outcome assessment (health care)"/ or treatment outcome/ or "process assessment (health care)"/ or program evaluation/ or quality assurance, health care/ or "patient acceptance of health care"/ or

needs assessment/ or health promotion/ or "Health Services Needs and Demand"/ or Preventive Health Services/ or Goals/ or Social Adjustment/

A2 – Keyword Searches

Population:

(aged or frail* or elder* or Geriatric* or (old* adj2 (person* or people)) or veteran* or octogenarian* or nonagenarian* or centenarian* or gerontol*).mp.

Assessment, Aftercare, Programs:

((aged or frail* or elder* or Geriatri* or (old* adj2 (person* or people or patient* or client*)) or veteran* or octogenarian* or nonagenarian* or centenarian* or gerontol* or patient*) adj3 (Assess* or evaluat* or manag* or apprais* or function* or Health Service* or aftercare or after-care or acute care or acute-care or comprehensive* or (patient* adj3 plan*) or (advance* adj3 plan*) or (progressive adj3 care) or patient care team or disability evaluation or patient* education or (Case adj3 Manag*) or manage* care program* or Critical Pathway* or Program* Evaluation or Health Education)).mp. OR (((consultative or comprehensive or evaluat*) adj3 geriatric* assessment) or (geriatric evaluati* adj3 management unit*) or stroke unit* or stroke team* or quality improvement program* or (geriatric* patient* adj3 care) or geriatric* inpatient* service* or early support* discharge* or intens* motor train* or comprehensive stroke unit* or (improve* adj3 program*) or orthogeriatric unit* or (hospital adj2 home) or hospital-at-home or multidisciplinary rehabilitat* or Motor Assessment Scale* or MAS or (Inpatient rehabilitat* adj3 unit*) or discharge destination* or (co?ordinated adj3 rehabilitat*) or (community?based adj3 rehabilitat*) or Early?supported discharge rehabilitat* or (reduce* length adj2 hospital*) or Post?discharge rehabilitat* or (transfer* adj3 (patient*

adj3 hospital*)) or (transfer* adj3 (patient* adj3 communit*)) or General practitioner?oriented or post?stroke rehabilitat* or hospital rehabilitat* or community rehabilitat* or (geriatric* evaluat* adj3 treat* unit*) or transition* care bridge or vulnerable elder* survey or geriatric* care program* or hospital elder life program* or (Acute Care adj3 Vulnerable Elder*) or (Nurse* Improv* Care adj3 Health?System Elder*) or (Hospital Elder Life Program) or (Nurses Improving Care to Health System Elders) OR (Program of All-Inclusive Care for the Elderly) or (Yale Geriatric Care Program)).mp.

OR ((Medicat* adj3 review*) or (rehabilitat* adj3 model*) or (inpatient* adj3 rehabilit*) or (dementia adj3 service*) or (care adj3 transition*) or (interdisciplinary adj3 transition*) or (nutrition* adj3 supplement*) or (pressure adj3 relie*) or ((elder* or geriatric* or senior*) adj3 (interven* or consult* or Prevent* or evaluat* or manag* or unit* or nursing or program* or Post-Acute Care* or acute care or section* or care unit* or care-unit* or Treatment* or Integrated service* or resource* or assess*)) or (multicomponent hospital-based intervention* or Psychogeriatric) or (Biopsychosocial adj3 (Evaluat* or Treatment* or Program*)) or Hospital in the Home* or translating research into practice* or ((Geriatric* Resource* adj2 (Assess* adj3 Care)) and Elders*) or ((Reduc* adj3 Fall*) and Elder*) or interdisciplinary comprehensive geriatric* assessment* or Senior care unit*).mp.

OR (Mobile geriatric* or geriatric* team* or (geriatric* and float* and interdisciplinary and transition and team*) or comprehensive geriatric* intervention* or acute geriatric* unit* or geriatric intervene* or (acute care adj3 elder*) or geriatric consult* team* or

(integrated care adj3 elder*) or geriatric* rehabilitation or (multidisciplinary adj3 care)).mp.

Hospital Units:

(acute*adj2 care* or emerg* or ((Emergenc* or hospital* or veteran* or trauma* or health* or care or surveillan* or coronary* or cardiac* or Intensive* or recover* or special* or geriatr* or elder* or aged* or operat* or respirat* or rehabilitat* or surg* or post-surg* or general* or medical* or inpatient* or in-patient*) adj3 (room* or department* or unit* or ward* or service* or care* or facilit*))).mp.

Cost and Cost Analysis:

((multi* adj3 (fact* or compo* or facet*)) or efficien* or reinforc* or cost-effect* or cost* or utilit* or economic* or nursing-led or interdisciplin* or multidisciplin* or pharmac* or inter-disciplin* or multi-disciplin* or social work* or social-work* or physic* therap* or physio-therap* or physical-therap* or occupational therap* or occupational-therap* or strateg* or best practic* or outcom* or quality of life or quality-of-life or adaptat* or success* or prepar* or method* or manag* or benefit* or plan* or coach* or innovat* or initiat* or influenc* or alternat* or promot* or integrat* or disseminat* or adopt* or occur* or prevail* or preval* or follow-up* or follow* up* or result* or servic* or restor* or implication* or detect* or enrich* or promot* or incident* or train* or collaborat* or measur* or rate* or rati* or control* or develop* or feasibl* or feasibil* or evaluat* or regimen* or reduc* or benefit* or interven* or design* or redesign* or re-design* or method* or inciden* or recover* or protect* or coordinat* or co-ordinat* or progres* or chang* or early or earlier or timing or time* or transition* or function* or Supplement* or Minimiz* or minimis* or design* or establish* or servic* or

support* or supplement* or screen* or discharg* or compar* or advis* or advic* or teach* or taught or learn* or follow-up or follow* up).mp.

Rehabilitation:

(Rehabilitat* or activities of daily living or early ambulat* or (exercise* adj2 therap*) or occupational therap* or (treatment adj2 outcome*) or physical therap* or exercise* movement technique* or breathing exercise* or motion therap* or muscle stretch* or resistance train* or music therap* or recovery of function* or exercise* or Self care* or medication therap* or Nutrition therap* or diet therap* or nutritional support* or enteral nutrition* or Parenteral Nutrition* or nutrition assessment* or Nutritional Status* or Fluid therap* or Accident prevent* or safety or primary prevent* or secondary prevent* or tertiary prevent* or Patient care or aftercare or (critical adj3 (care or nurs* or period*)) or (intensive adj3 (care or nurs* or period*)) or (perioperative adj3 (care or nurs* or period*)) or (peri-operative adj3 (care or nurs* or period*)) or (intraoperative adj3 (care or nurs* or period*)) or (intra-operative adj3 (care or nurs* or period*)) or (post-operative adj3 (care or nurs* or period*)) or (postoperative adj3 (care or nurs* or period*)) or (pre-operative adj3 (care or nurs* or period*)) or (preoperative adj3 (care or nurs* or period*)) or night* care or night?care or (emergency adj3 nurs*) or (geriatric adj3 nurs*) or (operat* room* adj3 (nurs* or care)) or ((postanesthe* or post-anesthe* or postanaesthe* or post-anaesthe*) adj3 (nurs* or care)) or (patient adj3 (position* or mov* or lift*)) or Psychiatr* Nurs* or Physical Medicine or Primary Nurs* or ((Nurs* or interdisciplin* or multidisciplin* or inter-disciplin* or multi-disciplin*) adj3 (Team* or process* or plan* or assess* or process*)) or (Patient adj2 Plan*) or Social Work* or social-work* or Pharmac* or physician* or general practitioner* or Therapeutic* or

(Environment* adj2 Design*) or Toilet* or ((Self-Help* or self help*) adj3 Device*) or Wheelchair* or wheel-chair* or Hearing Aid* or hearing-aid* or (Protect* adj3 Device*) or Bandage*).mp.

OR (multi-state model* or ((structure* adj3 education*) and model*) or multi-component or (multi* adj3 component*) or hospital-base* or (hospital adj3 base*) or (stretch* adj3 exercise*) or (physical* adj3 psychosocial function*) or (support adj3 surface*) or pressure ulcer* or multi-factor* or multifactor* or (exercise adj3 base*) or fast track* or fast-track* or vulnerable or (disabilit* adj2 prevent*) or (quality improve* adj3 intervention*) or hospital-based or post-acute or ((diet* or nutrition*) adj3 (supplement* or support*)) or fall-prevent* or fall* prevent* or vitamin or ((one-time or teach-to-goal or education*) adj3 (intervention or program)) or quality improve* or (reduce* adj3 pain) or recovery or (function* adj3 assess*) or (diet* adj3 assistant*) or physiotherap* or (discharge adj3 destination*) or (length* adj3 stay*) or complication* or (multi-state adj3 model*) or ((multi adj3 state) and model*) or trunk control test or risk assess* or nursing interven* or (restrain* adj3 reduc*) or supplementation or (hospital and discharge plan*) or (repetitive task adj3 train*) or music or ((improve* adj3 function*) and ability*) or malnutrition or mattress* or relax* or reinforc* or ((reduce* adj3 function*) and decline*) or (function* adj3 decline*)).mp.

OR (treat* or effect* or effic* or prevent* or enhanc* or model* or remodel* or motivati* or function* or reduc* or organiz* or improv* or outcom* or therap* or evaluat* or valu* or interven* or comprehen* or rehabilitat* or train* or exercis* or Transition* or Detect* or Examin* or characteri* or target* or impact* or identif* or support* or compar* or supplement* or progress* or comprehen* or implement*).mp.

OR (((massage or physi* or occupation*) adj3 therap*) or postoperat* or discharg* plan*).mp.

Hospitalization and Discharge:

(discharg* or admit* or admis* or readmit* or readmis* or hospitaliz* or process* or fall-prevent* or fall* prevent* or approach* or multidisciplin* or multi-disciplin* or interdisciplin* or inter-disciplin* or benefit* or physical function* or recover* or motivation* or assist* or factor* or approach* or delay* or (support* adj3 discharg*) or post-discharg* or post discharg* or mobiliz* or mobilis* or program* or decreas* or supplement* or strateg* or optim* or assess* or multifactor* or multicompo* or multifacet* or multi-factor* or multi-compo* or multi-facet*).mp.

Delivery of Healthcare:

((((health* adj 2 care) or health-care or healthcare) adj3 (deliver* or distribut* or system* or activit* or non-clinical* or non clinical* or communit* or quality or standard* or excellence or outcome* or out-come or process*)) or (outcome* adj3 (assess* or stud* or measure* or process* or treat*))).mp.

[a] Search strategy for early discharge planning performed concurrently with four other intervention components of Acute Care for Elders (ACE) model.

[b] Current referred to June 2011.
