# Supplementary material for: Effectiveness of early discharge planning in acutely ill or injured hospitalized older adults: a systematic review and meta-analysis
Source: BMC Geriatr. 2013 Jul 6;13:70. doi: 10.1186/1471-2318-13-70 (PMC3707815; doi:10.1186/1471-2318-13-70)
Supplement: Additional file 2 — Search Strategy on Specific Programs for MEDLINE(OVID). [file 1471-2318-13-70-S2.pdf]

<u>Additional File 2. Search Strategy on Specific Programs for MEDLINE(OVID)</u>

Limits applied:

Published Date:	yr="1985 -Current" [b]
Age Groups:	"all aged (65 and over)"
Languages:	English or French
Organism:	humans
Study Types:	case reports or clinical trial, all or clinical trial or comparative study or controlled clinical trial or meta analysis or multicenter study or randomized controlled trial or "review"

(Intermediate care or Transitional Care Packages or Payment of completed medical treatment or Transitional Care Model or Project BOOST Society of Hospital Medicine or Quality Initiatives for Hospitalized Patient Care or Re-engineered Discharge or Care Transitions Intervention or Transforming Care at the Bedside).mp.
