# Supplementary material for: Effectiveness of early discharge planning in acutely ill or injured hospitalized older adults: a systematic review and meta-analysis
Source: BMC Geriatr. 2013 Jul 6;13:70. doi: 10.1186/1471-2318-13-70 (PMC3707815; doi:10.1186/1471-2318-13-70)
Supplement: Additional file 3: Table S1 — Descriptive characteristics of studies included in systematic review and meta-analysis. Notes: a Not all outcomes measured at all time points. Where outcomes were comparable, narrative analysis or meta-analysis performed; b Duration of follow-up post index hospital discharge. Abbreviations: CHF = congestive heart failure; HT = hypertension; MI = myocardial infarction; N = sample size; NR = not reported; RCT = randomized controlled trial; USA = United States of America. [file 1471-2318-13-70-S3.pdf]

Additional File 3: Table S1. Descriptive characteristics of studies included in systematic review and meta-analysis

Study	Design	Setting/Target population	Co-morbidities	N	Age	Female gender (%)	Living arrangements	Outcomes assessed[a]	Time of assessment[b]
Choong [27]	Pseudo-RCT	Australia; all patients admitted through emergency department for surgical treatment of acute fracture of the femur.	NR	111	81	72%	Most (n = NR) lived with family or significant others.	Index length of hospital stay; hospital readmissions; time to surgery; time to mobilization; time to aged care assessment for discharge placement; discharge	Index hospital admission; daily; 28 days after index hospital discharge.

								destination; hospital complications; post-discharge complications.	
Kennedy [28]	RCT	USA; English speaking patients aged 75+ reachable by telephone.	NR	80	80	53%	Most (n = NR) lived with family or significant other in the community; community dwellers (96%); nursing home residents	Index length of hospital stay; hospital readmissions; mortality; time to first hospital readmission; change in living arrangement.	Index hospital admission; 2 and 8 weeks after index hospital discharge.

							(4%).		
Kleinpell [29]	RCT	USA; critically ill English speaking adults aged 65+ admitted to an intensive care unit for at least 24 hours and cognitively able to provide consent.	Cardiovascular 49%, oncological 21%, neurological 9%, hepatic or pancreatic 6%, and other 12%.	100	73	34%	NR.	Index length of hospital stay; quality of life; readiness for discharge.	Day before anticipated discharge from hospital; 2 weeks after index hospital discharge.
Legrain [34]	RCT	France; French speaking adults aged 70+ admitted to an	Coronary artery disease 29%, HT 66%, arrhythmia	665	86	66%	Living alone (47%).	Hospital readmissions; emergency department	Index hospital discharge; 3 and 6

	acute geriatric unit for emergency treatment of a medical condition. Excluded patients with an expected length of stay < 5 days, expected survival < 3 months, receiving palliative care, without health	25%, dementia 22%, diabetes mellitus 16%, heart failure 15%, cancer 13%, pulmonary disease 12%, and stroke 1.4%.			visits; mortality; delay before readmission.	months after index hospital discharge.

		insurance, not residing in France.							
Lin [30]	Quasi-experimental	Taiwan; adults aged 65+ with hip fracture; able to walk; pre-fracture Barthel score ≥ 70 points; not cognitively impaired; not terminally ill.	NR	50	79	36%	NR	Index length of hospital stay; hospital readmissions; satisfaction with discharge planning; quality of life; self-care knowledge; functional status.	Index hospital admission and discharge; 2 and 12 weeks after index hospital discharge.

Naughton [33]	RCT	USA; Adults aged 70+ admitted from emergency department to medical care and not under care of hospital staff.	NR. Most frequent admitting diagnoses included CHF 8%, pneumonia 7%, syncope 5%, urinary tract infection 5%.	111	80	57%	Living at home (78%).	Index length of hospital stay; discharge destination; costs.	Index hospital discharge.
Naylor [23]	RCT	USA; English speaking adults aged 65+ admitted from their homes with a	Coronary artery disease 48%, HT 52%, atrial tachycardia 35%, diabetes mellitus 38%,	239	76	57%	NR other than admitted from their homes.	Index length of hospital stay; hospital readmissions or death after discharge;	Index hospital admission; 2, 6, 12, 26, and 52 weeks after

	diagnosis of heart failure; not diagnosed with end-stage renal disease; alert and oriented; reachable by telephone after discharge, and residing within 60 miles from hospital.	and pulmonary disease 30%.		readmission length of hospital stay; mortality; satisfaction with discharge planning; quality of life; functional status; costs; time to first hospital readmission or death; unscheduled acute care	index hospital discharge.

								visits.	
Rich [32]	RCT	USA; adults aged 70+ admitted to medical unit with CHF, residing within catchment area, not planned to be discharged to nursing home or chronic care facility, without severe cognitive	HT 65%, diabetes mellitus 31%, prior MI 23%, prior CHF 63%.	98	79	59%	NR; Living in community presumed because excluded patients with planned nursing home discharge.	Index length of hospital stay; hospital readmissions; readmission length of hospital stay; time to first hospital readmission.	Index hospital discharge; 90 days after index hospital discharge.

		impairment, psychiatric disturbance, or non-cardiac illness with probability of unpreventable readmission.							
Rich [31]	RCT	USA; adults aged 70+ admitted with CHF; one or more risk factors for readmission (history of	HT 76%, diabetes mellitus 28%, prior MI 43%, prior CHF 77%.	282	79	59%	Living alone 43%; living with others 57%; living in community presumed because planned	Hospital readmissions; readmission length of hospital stay; mortality; quality of life; costs.	Index hospital admission; 90 days after index hospital discharge.

	heart failure, > 3 hospitalizations within past 5 years, or CHF precipitated by acute MI or uncontrolled HT); living within the catchment area; not planned to be discharged to a long term care facility; no severe	nursing home discharges excluded.

	dementia; no psychiatric illness; expected survival > 3 months.	

Notes: [a] Not all outcomes measured at all time points. Where outcomes were comparable, narrative analysis or meta-analysis performed; [b] Duration of follow-up post index hospital discharge.

Abbreviations: CHF = congestive heart failure; HT = hypertension; MI = myocardial infarction; N = sample size; NR = not reported; RCT = randomized controlled trial; USA = United States of America
