# Supplementary material for: Effectiveness of early discharge planning in acutely ill or injured hospitalized older adults: a systematic review and meta-analysis
Source: BMC Geriatr. 2013 Jul 6;13:70. doi: 10.1186/1471-2318-13-70 (PMC3707815; doi:10.1186/1471-2318-13-70)
Supplement: Additional file 5: Table S2 — Characteristics of early discharge planning intervention and usual care. Abbreviations: ADL = activities of daily living; APN = advanced practice nurse; CNS = clinical nurse specialist; GEM = geriatric evaluation and management; IADL = instrumental activities of daily living; ICU = intensive care unit; NR= not reported; PT = physical therapist; SW = social worker. [file 1471-2318-13-70-S5.doc]

| **Additional File 5: Table S2. Characteristics of early discharge planning intervention and usual care** | | |
| --- | --- | --- |
| Study | **Early discharge planning intervention** | Usual care |
| Choong [27] | ***Initiated*: on index hospital admission.**  ***Duration*: NR; index length of hospital stay presumed.**  ***Included*: Clinical pathway that comprised early ambulation; pathway documentation; orthopedic consultation; individualized discharge planning based on baseline level of independence; and provision of information on wound care, expected milestones, contact details, and simple exercises.**  *Provided by*: multidisciplinary team. | *Initiated*: post-operatively.  ***Duration*: NR; index length of hospital stay presumed.**  ***Included*: 48 hour postoperative bed rest, ad hoc documentation, individualized discharge planning based on patient progress, and provision of** discharge phone call and summary to discharge destination.  *Provided by*: multidisciplinary team. |
| Kennedy [28] | ***Initiated*: “early” on admission.**  ***Duration*: after index hospital discharge but length of duration NR.**  ***Included*: comprehensive physical, cognitive, and psychosocial nursing assessment. Identification of discharge resources and support networks. Development, implementation, and documentation of comprehensive discharge planning protocol based on assessments. Family, patient, and healthcare team meeting. Patient and family discharge planning teaching to clarify discharge plans. Daily follow-up to assess possibility of discharge. Follow-up visit after index hospital discharge.**  *Provided by*: **Gerontological CNS with assistance of other healthcare team members.** | ***Initiated*: NR**  ***Duration*: NR; index length of hospital stay presumed.**  ***Included*: individualized planning based on post-hospital needs. Support, teaching and referral needs documented in medical record.**  *Provided by*: primary nurses with assistance of other healthcare team members. |
| Kleinpell [29] | ***Initiated*: within 24 to 48 hours of index hospital admission, while in ICU.**  ***Duration*: index length of hospital stay.**  ***Included*: ICU based screening intervention to determine discharge needs. Areas of assessment included: patient perception of anticipated home needs after index hospital discharge, ADL, IADL, environment, social support, and patient preference for discharge preparations. Assessment results formally communicated to discharge planning nurse at time of transfer from ICU.**  *Provided by*: nurse. | ***Initiated*: 1 to 3 days before index hospital discharge, after transfer from ICU.**  ***Duration*: index length of hospital stay.**  ***Included*: general unstructured discharge planning or discharge follow-up on specific referral by physician, nurses or social worker.**  *Provided by*: NR. |
| Legrain **[34]** | ***Initiated*: “early” after admission.**  ***Duration*: NR; index length of hospital stay presumed.**  ***Included*: usual care; screening for depression and malnutrition; medication review and adjustment; patient and/or caregiver education focused on disease management; comprehensive discharge planning focused on community healthcare provider involvement including community physicians to obtain agreement with treatment plan, and to plan and transfer care which may have included follow-up home visits or telephone calls.**  *Provided by*: intervention dedicated geriatrician. | ***Initiated*: NR.**  ***Duration*: NR; index length of hospital stay presumed.**  ***Included*: comprehensive geriatric assessment; no consistent involvement of community physician; no specific education focused on disease management.**  *Provided by*: multidisciplinary team. |
| Lin [30] | ***Initiated*: within 48 hours of index hospital admission.**  ***Duration*: 3 months after index hospital discharge.**  ***Included*: comprehensive nursing discharge planning needs’ assessment; individualized patient and caregiver education based on needs; service monitoring; resource coordination; referral placement arrangement; 2 home visits for support and consultation at 2 weeks and 3 months after index-hospital discharge.**  *Provided by*: nurse. | ***Initiated*: NR.**  ***Duration*: NR; index length of hospital stay presumed.**  ***Included*: routine discharge nursing care that may have included discharge instructions in accordance with individual nurses’ judgment.**  *Provided by*: nurse. |
| Naughton [33] | ***Initiated*: upon index hospital admission.**  ***Duration*: 2 weeks after index hospital discharge.**  ***Included*: comprehensive assessment; geriatrician services that included treating medical condition, adjusting medications, overseeing rehabilitation, referring to psychiatry; discharge planning that included team conferences 2 to 3 times/week during which the patient’s living situation, social supports, family and patient educational needs, and patient progress were reviewed, arrangement of home care services, provision of caregiver support and education, coordination of transfer to home health care or to interdisciplinary outpatient care program for patients with no primary care provider, and follow-up to ensure post-discharge plans were in place.**  *Provided by*: **GEM team that included SW and geriatrician in collaboration with physicians, CNS and PT.** | ***Initiated*: NR.**  ***Duration*: NR; index length of hospital stay presumed.**  ***Included*: usual care with discharge planning upon referral.**  *Provided by*: physicians **with SW and discharge planner upon referral.** |
| Naylor [23] | ***Initiated*: within 24 hours of index hospital admission.**  ***Duration*: 3 months after index hospital discharge.**  ***Included*: comprehensive assessment, discharge planning, and arrangement of home care services focused on preventing functional decline and streamlining medication regimes. Identification of patient and caregiver goals; development of individualized care plans; patient and caregiver teaching; daily APN visits during index hospital stay and re-hospitalizations (where applicable); care continuity and coordination across settings; transfer of information, including medications; post-discharge APN in-home patient assessment within 24 hours of index hospital discharge; APN in-home visits every week during first month, and every 2 weeks during the second and third month of index hospital discharge; daily APN telephone availability.**  *Provided by*: **APNs in collaboration with physicians, nurses, and discharge planners.** | ***Initiated*: NR.**  ***Duration*: NR but may have been continued after index hospital discharge (**58% received nursing or physical therapy referrals upon index hospital discharge)**.**  ***Included*: routine hospital care including heart-failure management, discharge planning, and standard home care if referred.**  *Provided by*: nurses, physicians, and discharge planners. |
| Rich [32] | ***Initiated*: “early” after index hospital admission**  ***Duration*: 90 days after index hospital discharge**  ***Included*: patient education about condition, treatments including medications and diet; documentation and review of medications with recommendations to improve adherence and reduce side effects; early consultation with social services to facilitate discharge planning; completion of discharge summary indicating medication, diet, activity, and potential problem areas; daily visits by cardiac nurse during hospitalization; follow-up home visit within 48 hours of index hospital discharge and telephone contacts; and encouragement to contact study nurse if required.**  *Provided by*: **study nurses, geriatric cardiac nurse, SW, dietician, geriatric cardiologist, home-care team member.** | ***Initiated*: NR.**  ***Duration*: NR.**  ***Included*: conventional treatments, prescribed by physician, which may have included social service evaluation, patient education, and home care (34% received home care referrals).**  *Provided by*: NR. |
| Rich [31] | ***Initiated*: “early” after index hospital admission.**  ***Duration*: 90 days after index hospital discharge.**  ***Included*: patient education about condition, treatments including medications and diet; documentation and review of medications with recommendations to improve adherence and reduce side effects; early consultation with social services to facilitate discharge planning; completion of discharge summary indicating medication, diet, activity, and potential problem areas; follow-up via home visit within 48 hours of index hospital discharge and via telephone; and encouragement to contact study nurse if required.**  *Provided by*: **nurses in collaboration with geriatric cardiac nurse, social worker, dietician, geriatric cardiologist, home-care team member.** | ***Initiated*: NR.**  ***Included*: standard treatment and service prescribed by physician.**  ***Duration*: NR.**  *Provided by*: NR. |

**Abbreviations:** **ADL = activities of daily living; APN = advanced practice nurse;** CNS = clinical nurse specialist; GEM = geriatric evaluation and management; **IADL = instrumental activities of daily living;** **ICU = intensive care unit;** NR = not reported; PT = physical therapist; **SW = social worker.**
